# Capillary Dentures

**Published:** 1884-05

**Authors:** C. H. Land

**Affiliations:** Detroit, Mich.


### CAPILLARY DENTURES.

BY DR. C. H. LAND, DETROIT, MICH.

It is a common belief among dentists that artificial teeth are maintained in the mouth by atmospheric pressure, and the thousands of abominable air spaces to be seen in every-day practice, even among some of the most prominent practitioners, are such positive illustrations of ignorance as to call forth the most severe censure. The motives that prompt a man to create an air space, always the same size and in the same place, the same depth, and in every mouth just alike, must be imagined; they cannot be comprehended.

In a paper entitled "Atmospheric Dentures," which was read before the Michigan Dental Association at its last annual meeting, held March 26, 1884, I endeavored to show the relation of the atmosphere to artificial dentures as serving but a temporary means to establish an adaptation, and this only where an air space had been provided, the tongue being the exhausting medium. Air pressure cannot exist without the air space and a pump to exhaust it. Yet you will hear dentists talking about fifteen pounds' pressure to the square inch in the mouth.

Capillary attraction is the force that causes the denture to adhere. When two plane surfaces are placed close to each other, and brought in contact with any fluid substance, the force of capillary attraction is manifested. The moisture enters into and between the surfaces, going against its own gravity, causing the plates to unite with considerable force, and, although the atmosphere has a tendency to occupy space with a force equal to fifteen pounds to the square inch, the moisture has driven it out, and occupied the space originally held by it. At this juncture we have arrived at a state of equilibrium, having no air space, no vacuum, consequently no pressure, and if these latter terms were left entirely out of consideration, it would be much better for both the operator and his patient.

In preparing a denture it is simply necessary to make a suitable relief for the rigid portions of the mouth, especially the palatine surface, by using a thin piece of tin or lead covered with tin-foil, in thickness not more than the sixty-fourth part of an inch. This should cover at least four-fifths of the lingual surface. By this means a partial vacuum is unavoidable, until the tissues and fluids of the mouth fill it so completely as to shut out all air spaces, and until this point is reached there cannot be a perfect adaptation.

Therefore, if we pay more attention to augmenting the moist condition, which is the real adapting medium, and a proper system of relief, such expressions as air chamber, air pressure, and vacuum, will be meaningless.

